# Effects of Barbell Squats with Asymmetric Loading on the Joint Moment and Muscle Activity of Lower Limbs

**DOI:** 10.5114/jhk/202020

**Published:** 2025-09-23

**Authors:** Peirong Liu, Yongjie Li, Boya Zhang, Wenqiang Weng, Duo Li, Yong Ma, Weitao Zheng

**Affiliations:** 1 Key Laboratory of Sports Engineering of General Administration of China, Wuhan Sports University, Wuhan, China.; 2 Research Center for Sports Engineering and Health Intelligent Equipment of Hubei Province, Wuhan Sports University, Wuhan, China.; 3 Department of Rehabilitation, Beijing Jishuitan Hospital Guizhou Hospital, Guiyang, China.

**Keywords:** asymmetric loading, offset load training, joint moment, muscle activity, barbell squat

## Abstract

This study aimed to investigate the effects of barbell squats with asymmetric loading on bilateral joint moments and muscle activity of the lower limbs. Twenty fitness athletes were recruited to perform squats under five different conditions. The peak moments in the sagittal plane of the hip and knee joints and the root mean square (RMS) of the gluteus maximus, rectus femoris, and semitendinosus muscles were analyzed bilaterally in the lower limbs, and symmetry was assessed bilaterally using the symmetry index (SI). In the non-dominant side 10% offset load (10%NDOL), the difference in the RMS and moments between the dominant and non-dominant sides was not significant (p > 0.05). Compared with the symmetrical load (SL), the hip and knee SI_M_, gluteus maximus, rectus femoris, and semitendinosus SI_RMS_ were significantly lower on the non-dominant side 5% offset loads (5%NDOL) and 10% NDOL (p < 0.05), and the 10% offset load compared to the 5% offset load difference was significantly lower (p < 0.01). The NDOL reduced the differences in joint moments and muscle activity bilaterally in the lower limbs, with a 10% offset load being more favorable, and the limb SI was significantly negatively correlated with the amount of the offset load in the NDOL. The non-dominant side offset squats are beneficial for balancing muscle strength bilaterally in the lower limbs and improving bilateral strength asymmetry.

## Introduction

Barbell squats are among the most common exercises used by athletes and fitness enthusiasts to build muscle size, strength, and power in the lower extremities (Coratella et al., 2019; Kubo et al., 2019; Marián et al., 2016; van den Tillaar et al., 2024). The traditional barbell squat exercise places symmetrical loads bilaterally on the barbell to perform the exercise and is normally considered an almost symmetrical task (Escamilla, 2001; Kobayashi et al., 2010; Suchomel et al., 2024). However, bilateral asymmetries are present in joint moments of the lower extremities during barbell squats using symmetrical loads (Flanagan and Salem, 2007; Kobayashi et al., 2010). Previous findings suggest that bilateral strength asymmetry in the lower limb negatively affects athletic performance (Hart et al., 2014; Turkeri et al., 2024) and is associated with injury (Chavarro-Nieto et al., 2023; Croisier et al., 2008); therefore, there is a need for targeted measures to minimize the extent of bilateral strength asymmetry.

Human limbs exhibit different levels of individual limb strength for several reasons (Sato and Heise, 2012; Schmid et al., 2010). In barbell squats, asymmetry in bilateral strength results in a shift of the center of gravity to one side, or the barbell is unbalanced (where one side is lifted higher than the other), which leads to overloading of one of the limbs, resulting in an increased bilateral difference in strength (Sato and Heise, 2012). Therefore, symmetrically loaded barbell squats may not be an effective way to maintain bilateral lower extremity symmetry, and additional techniques may be required for optimal training (Flanagan and Salem, 2007; Kobayashi et al., 2010).

Asymmetric loading training, also known as offset training, is a recently proposed strategy for reducing limb strength asymmetries, in which asymmetric loading involves loading one side of a resistance implement (barbell) to a greater or lesser degree than the other side, thus creating an asymmetric load between limbs during bilateral exercises. Bilateral asymmetric loads applied during barbell bench press exercises lead to differences in muscle activity between the dominant and non-dominant sides (Jarosz et al., 2020; Saeterbakken et al., 2020). In addition, previous research assessed pectoral muscle thickness by ultrasonography and muscle strength by bench press one-repetition maximum, and has shown that a four-week mesocycle of the offset loading barbell bench press is more effective in enhancing adaptations in hypertrophy and strength than the traditional loading barbell bench press (Sharp, 2022). Asymmetry in strength on both sides of the limb can be counteracted as the volume of the workout increases (Jarosz et al., 2020; Saeterbakken et al., 2020). However, when bilateral asymmetric loading is applied to the barbell squat, only changes in the bilateral limb ground reaction forces are observed. The muscle activation resulting from asymmetric loading that occurs in the barbell bench press was not observed in the barbell squat, which poses a challenge for offset training (Ottinger et al., 2023). Nevertheless, research on asymmetric loading barbell squats still requires examination of the amount of offset loading, as this may be responsible for the differences in the unstable environment in which the bilateral exercise is performed. As found in unilateral training and other exercises performed under unstable conditions, a greater degree of instability may be necessary for further adaptations, especially in well-trained populations (Behm and Colado, 2012; Behm et al., 2010; Wahl and Behm, 2008). In addition, the effects of the external structures of asymmetrically loaded exercises, such as the joint moment, should be examined to explore the differences in musculoskeletal dynamics during exercise (Escamilla, 2001; Schoenfeld, 2010). Therefore, this study aimed to investigate the effects of barbell squatting with asymmetric loading on the joint moments and muscle activities of the lower limbs bilaterally under the same total load but different offset loads. We hypothesized that the non-dominant side offset load would reduce the differences in the joint moment and muscle activity bilaterally in the lower limbs, and the differences would decrease as the offset load increased.

## Methods

### Participants

The predicted sample size was calculated using G*Power 3.1. Analysis was performed using a 0.3 effect value, and at a significance level of α = 0.05 and a statistical power of 80%, the results showed that 18 participants were needed (Faul et al., 2007). Considering a sample dropout rate of 10%, 20 participants were recruited for this experiment. The inclusion criterion was no major lower extremity neuromuscular injury within 6 months before the experiment. All participants were experienced fitness athletes and had more than 3 years of training experience, with an expected barbell squat one-repetition maximum strength test (1RM) > 1.5 × body weight. Before the start of the experiment, all participants were informed verbally and in writing about the study procedure and they completed an informed consent form. This study was approved by the Wuhan Sports University Medical Ethics Committee, Wuhan, China (approval code: 2023052; approval date: 05 July 2023). Table 1 summarizes the baseline characteristics of the participants.

**Table 1:** Characteristics of participants.

Age (yrs)	Body height (cm)	Body mass (kg)	Dominant side	Leg length (cm)	Pelvic width (cm)
20.85 ± 1.55	178.57 ± 5.77	76 ± 5.32	Right	91.3 6 ± 4.05	28.31 ± 2.01

### Study Design

The barbell squat 1RM test was performed first, and participants were familiarized with an asymmetrically loaded barbell squat (Grgic et al., 2020). In addition, the dominant and non-dominant sides were differentiated by the preferred kicking leg (van Melick et al., 2017). Tests were performed one week apart, with participants performing barbell squats under different conditions of the symmetrical load (SL), the dominant side 5% offset load (5%DOL), the non-dominant side 5% offset load (5%NDOL), the dominant side 10% offset load (10%DOL), and the non-dominant side 10% offset load (10%NDOL). Different loading conditions were set randomly, and 5% and 10% of 60% 1RM were calculated and used as the difference in loading between the two sides of the barbell. When a participant had 1RM of 100 kg, the load difference between the two sides of the barbell would be 0 kg (SL), 3 kg (5%), and 6 kg (10%). All the offset loads were adjusted to the nearest 0.5 kg. Mean offset loads were 5.65 ± 0.78 kg (5%) and 10.32 ± 1.69 kg (10%).

### Electromyography (EMG)

Changes in the EMG signals during the participant's movements were recorded using a surface EMG system (Noraxon, Telemyo2400DTS, USA, 1.5 kHz). The gluteus maximus, semitendinosus, and rectus femoris were tested on both sides of the body. The attachment and fixation of the electrode pads were performed by the same experimenter for all participants. The participants were first tested for the maximum voluntary contraction of the muscles to normalize the EMG signals collected during subsequent exercise conditions (Shibata et al., 2021). The tests were performed in randomized order, and the participants were required to contract the resistance with maximum force and hold it for 5 s. Each muscle was tested three times, with a 30-s rest interval in between. After testing, the Vicon system was synchronized with the Kistler system using a digital signal converter. Kinematic, kinetic, and EMG data were collected during the participants’ testing using port commands and synchronization cables to synchronize Vicon with the Noraxon system.

### Sports Data Collection

**Figure 1:**
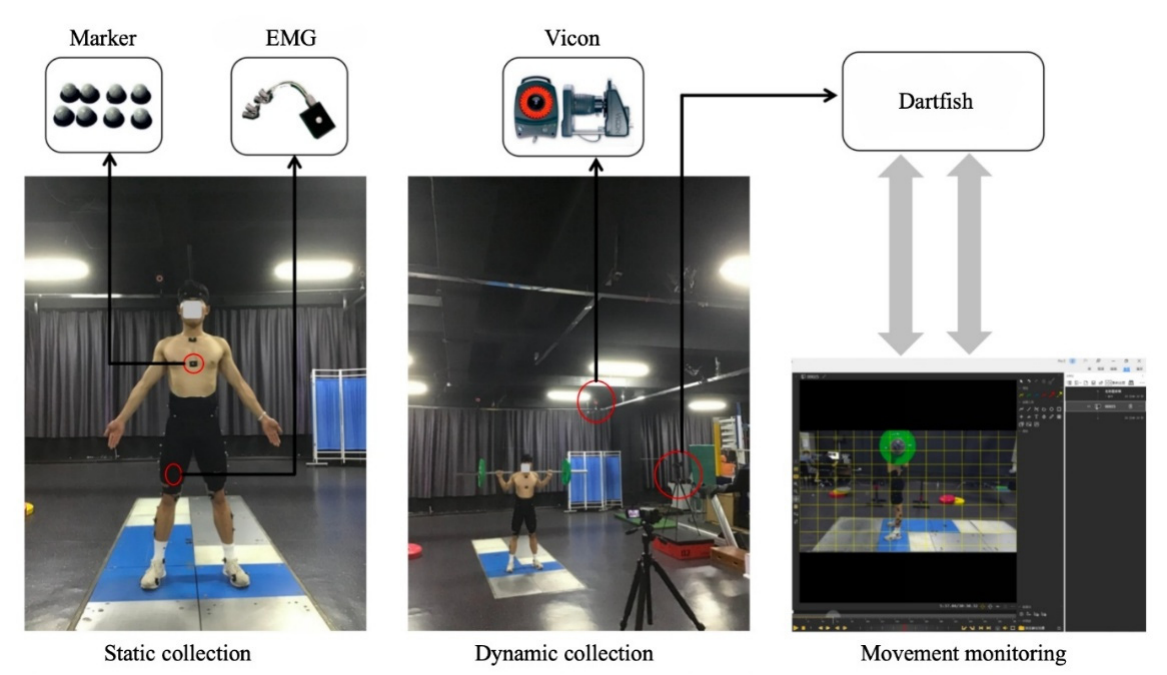
Experimental site.

The kinematic data were acquired using a motion capture system (Vicon, model T40, UK; 200 Hz). Kinetic data were acquired using a force plate (Kistler Model 9260AA6, Switzerland; 1 kHz). A total of 46 markers were pasted by the same experimenter based on the participants' bone markers and tracking points. Participants completed the asymmetrically loaded barbell squat under movement monitoring; 12 repetitions were performed with a 90-s rest interval scheduled between groups, and the mean values during the repetitive lifting period were used (Saeterbakken et al., 2020). The standardized movement consisted of hands slightly wider than shoulder width, with the barbell placed over the posterior deltoid fascicle. During the descent, the lowest point was at the thighs, parallel to the floor. During the process, the spine and pelvis were kept stable and balanced, and the movement was performed in a 2-s uniform speed squat to the bottom without stopping, followed by a 2-s uniform speed rise. The quality of the movements was monitored in real-time by an on-site senior physical trainer using Dartfish software (Lu et al., 2020). The experimental site is shown in Figure 1.

### Data Processing

The collected data were matched to the self-constructed model using Vicon software and imported into Visual3D (C-Motion, USA) in the C3D format for calculation. Using the inverse dynamics method, the joint moments were defined as the moments of the distal segments relative to the proximal phase. The data were filtered using a low-pass 10-Hz (25 Hz) filter and normalized to the body mass of each participant. The peak sagittal moments (M) in the hip and knee joints during the centrifugal phase of the movement were calculated for analysis.

The raw surface EMG data were rectified, smoothed, filtered (band-pass filtered 20–400 Hz), and amplitude-normalized using the Noraxon software. The EMG root mean square (RMS) was calculated for each muscle during the centrifugal phase of movement to analyze the changes in muscle activity.

To analyze bilateral differences in the limbs, limb symmetry was assessed using the symmetry index (SI) (Arhos et al., 2021; Nelson et al., 2018) calculated with formulas (1) and (2).


$${\text{SI}}_{M}=\left|\frac{M\text{DS}-M_{NDS}}{M_{DS}+M_{NDS}}\right|\times 100\%$$

$${\text{SI}}_{RMS}=\left|\frac{RM{\text{S}}_{\text{DS}}-RMS_{NDS}}{RMS_{DS}+RMS_{NDS}}\right|\times 100\%$$

where M represents the peak joint moment, DS represents the dominant side, and NDS represents the non-dominant side.

### Statistical Analysis

The normal distribution test was performed following the Shapiro-Wilk method, which showed that the data conformed to a normal distribution, and the data were statistically analyzed using SPSS 26.0 and expressed as mean ± standard deviation (M ± SD). Differences in the lower limb joint moments and the muscle RMS across the side and load conditions were analyzed using a 2 × 5 (side × load condition) repeated- measures ANOVA. Differences in the limb SI across sides and load conditions were analyzed using one-way repeated-measures ANOVA. Post-hoc tests were performed applying the Bonferroni correction, and the level of significance was set at *p* < 0.05. In addition, the Spearman's correlation coefficient was calculated to assess the relationship between the offset load and the limb SI in both the NDOL and DOL conditions.

## Results

### Joint Moments

**Figure 2:**
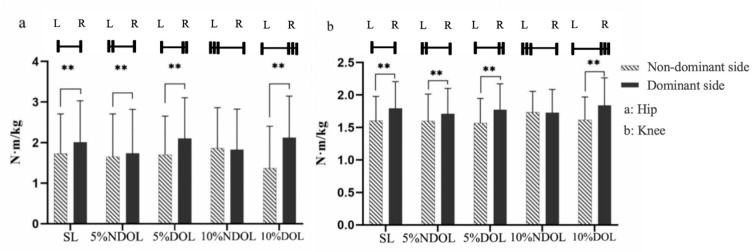
Joint moments under different lateral and load conditions. SL, 5% NDOL, 5% DOL, 10% NDOL, and 10% DOL represent symmetrical loads, non-dominant side 5% offset load, dominant side 5% offset load, non-dominant side 10% offset load, and dominant side 10% offset load, respectively; a and b represent the results of the hip and the knee, respectively; * p < 0.05, ** p < 0.01

The results showed that different side and loading condition interactions had significant effects on both hip and knee joint moments [hip: (*p* < 0.01; *F* = 252.70; *η^2^* = 0.91); knee: (*p* < 0.01; *F* = 19.55; *η^2^* = 0.45)]. The results of the simple effects analysis are shown in Figure 2. The dominant side was significantly higher than the non-dominant side in both the SL, 5%DOL, and 10%DOL groups (*p* < 0.01). However, in the 10%NDOL group, the difference between the dominant and non-dominant sides was not statistically significant (*p* > 0.05). In addition, the differences in joint moments between the dominant and non-dominant sides under different loading conditions were not statistically significant (*p* > 0.05).

### RMS

**Figure 3:**
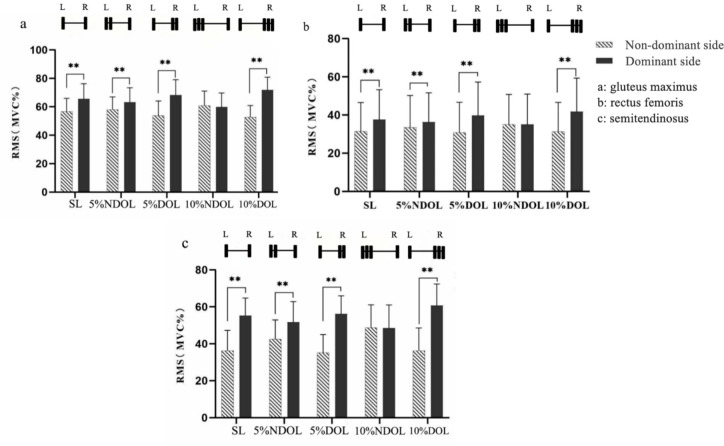
RMS under different lateral and loading conditions. SL, 5% NDOL, 5% DOL, 10% NDOL, and 10% DOL represent symmetrical load, non-dominant side 5% offset load, dominant side 5% offset load, non-dominant side 10% offset load, and dominant side 10% offset load, respectively; a, b, and c represent the results of the gluteus maximus, rectus femoris, and semitendinosus muscles, respectively; * p < 0.05, ** p < 0.01

The results showed that different side and load difference interactions had significant effects on the RMS in the gluteus maximus (*p* < 0.01; *F* = 216.29; *η^2^* = 0.90), rectus femoris (*p* < 0.01; *F* = 17.64; *η^2^* = 0.43), and semitendinosus (*p* < 0.01; *F* = 162.93; *η^2^* = 0.87) muscles. The results of the simple effects analysis are shown in Figure 3. The dominant side had a significantly higher RMS than the non-dominant side in the SL, 5%DOL, and 10%DOL groups (*p* < 0.01). However, in the 10%NDOL group, the difference between the dominant and non-dominant sides was not statistically significant (*p* > 0.05). In addition, none of the differences in muscle RMS comparisons between the dominant and non-dominant sides under different loading conditions were statistically significant (*p* > 0.05).

### Symmetry Index

As shown in Table 2, there were significant differences considering the hip and knee *SI_M_* under different loading conditions (*F* = 74.29, *p* < 0.01; *F* = 37.47, *p* < 0.01). Post-hoc tests showed that the 5%DOL and 10%DOL groups presented significantly higher values than the SL group (*p* < 0.05), whereas the 5%NDOL and 10%NDOL groups showed significantly lower values than the SL group (*p* < 0.05). The 10%NDOL group’s results were significantly lower than those of the 5%NDOL group (*p* < 0.01). In addition, the *SI_RMS_* values of the gluteus maximus, rectus femoris, and semitendinosus were significantly different under different loading conditions (*F* = 243.55, *p* < 0.01; *F* = 24.36, *p* < 0.01; and *F* = 107.64, *p* < 0.01, respectively). Post-hoc tests showed that the 5%NDOL and 10%NDOL groups had significantly lower values than the SL group (*p* < 0.05), whereas the 10%NDOL group presented significantly lower values than the 5%NDOL group (*p* < 0.01).

**Table 2:** Limb SI under different loading conditions.

	SL	5%NDOL	5%DOL	10%NDOL	10%DOL	*F* Test	
						***F***	***p***
Hip	8.70 ± 3.98	3.13 ± 2.39^*^	12.03 ± 4.18^*^	1.19 ± 2.04^*##^	27.95 ± 13.50^*##^	74.29	<0.01
Knee	5.49 ± 1.88	3.61 ± 2.31^*^	6.46 ± 3.14^*^	0.50 ± 2.61^*##^	6.27 ± 2.66^*##^	37.47	<0.01
Gluteus maximus	7.37 ± 1.46	4.20 ± 1.56^*^	12.07 ± 3.36^*^	0.81 ± 0.84^*##^	15.62 ± 2.50^*##^	243.55	<0.01
Rectus femoris	9.73 ± 5.15	5.49 ± 5.64^*^	14.38 ± 6.47^*^	0.12 ± 2.29^*##^	15.47 ± 11.06^*##^	24.36	<0.01
Semitendinosus	22.06 ± 7.65	10.06 ± 4.49^*^	23.92 ± 5.54^*^	0.33 ± 1.22^*##^	26.79 ± 8.19^*##^	107.64	<0.01

SL, 5% NDOL, 5% DOL, 10% NDOL, 10% DOL represent symmetrical load, non-dominant side 5% offset load, dominant side 5% offset load, non-dominant side 10% offset load, dominant side 10% offset load, respectively, Compared to symmetrical loads, ^*^ p < 0.05, ^**^ p < 0.01, Compared to 5% offset load, ^#^ p < 0.05, ^##^ p < 0.01

### Correlation Analysis

The correlation analysis results showed a significant correlation between the *SI_M_* and the amount of the offset load in the DOL (hip: r_s_ = 0.69, *p* < 0.01; knee: r_s_ = 0.58, *p* < 0.05; gluteus maximus: r_s_ = 0.81, *p* < 0.01; rectus femoris: r_s_ = 0.29, *p* < 0.05; and semitendinosus: r_s_ = 0.25, *p* < 0.05). *SI_RMS_* showed a significant association with the amount of the offset load in the NDOL (hip: r_s_ = −0.86, *p* < 0.01; knee: r_s_ = −0.75, *p* < 0.01; gluteus maximus: r_s_ = −0.91, *p* < 0.01; rectus femoris: r_s_ = −0.72, *p* < 0.01; and semitendinosus: r_s_ = −0.84, *p* < 0.01). The results are presented in Table 3.

**Table 3:** Correlation analysis between offset loads and the limb SI.

	DOL	NDOL
*SI_M_* (Hip)	0.69**	−0.86**
*SI_M_* (Knee)	0.58*	−0.75**
*SI_RMS_* (Gluteus maximus)	0.81**	−0.91**
*SI_RMS_* (Rectus femoris)	0.29*	−0.72**
*SI_RMS_* (Semitendinosus)	0.25*	−0.84**

DOL = dominant side offset load; NDOL = non-dominant side offset load; SI_M_ = limb symmetry index of joint moments; SI_RMS_ = limb symmetry index of the root mean square; * Correlation is significant (p < 0.05); ** Correlation is significant (p < 0.01)

## Discussion

This study aimed to investigate the effects of barbell squats with asymmetric loading on bilateral joint moments and muscle activity of the lower limbs. The results showed that in the SL and DOL, joint moments and muscle activities were significantly higher on the dominant than on the non-dominant side. However, in the NDOL, reduced differences in joint moments and muscle activities were observed bilaterally in the lower limbs compared to the SL, with the 10% offset load showing smaller values than the 5% offset load. In addition, both *SI_M_* and *SI_RMS_* were significantly lower for the NDOL than for the SL, with the 10% offset load presenting lower values than the 5% offset load. The limb SI was significantly negatively correlated with the amount of the offset load in the NDOL.

The RMS results of this study showed an interaction effect between the different sides and loading conditions, as demonstrated by a higher RMS on the dominant than on the non-dominant side in SL and DOL, whereas in NDOL, the difference between the bilateral RMS of the lower limbs decreased.

This suggests that the muscle activity of the lower extremities on that side increased when an offset load was applied to one side of the barbell. The results of this study are consistent with those of two previous offset load studies (Jarosz et al., 2020; Saeterbakken et al., 2020). However, similar results were not obtained in a previous study of offset loading in barbell squats (Ottinger et al., 2023). Ottinger et al. (2023) placed offset loads on the dominant and non-dominant sides, their study subjects performed barbell squats, and no asymmetries between the dominant and the non-dominant side muscle activations were found. A probable reason for this discrepancy is that the subjects in our study had large interlimb strength asymmetry at baseline. In addition, our study was set up with a larger offset load (our study: 5%–10%, Ottinger et al.’s study: 5%). During an asymmetrically loaded barbell squat, when one side of the barbell is loaded more than the other, a greater force is applied to one of the limbs, disrupting the balance of the body. This creates an unstable environment that allows for increased muscle activation and co-contraction on one side of the limb, resulting in increased activation of the antagonist muscles to enhance stability and maintain postural and body balance during movement (Baratta et al., 1988). In addition, the results showed that the *SI_RMS_* was significantly negatively correlated with the amount of the offset load in the NDOL. The *SI_RMS_* value decreased as the offset load increased. Specifically, gluteus maximus, rectus femoris, and semitendinosus muscles showed reduced values by 3.2%, 4.2%, and 12%, respectively, with 5% NDOL, whereas these values decreased by 6.6%, 9.6%, and 21.7%, respectively, with 10% NDOL. Surface electromyography (EMG) was used to show that higher external loads resulted in greater muscle activation than lower loads (Martinez et al., 2023; van den Tillaar et al., 2019). In the present study, it could be inferred that an increased load on one side of the barbell required greater muscle activation in the same limb. An increased offset load imposed a greater destructive torque on the body, making it more difficult for the body to maintain balance. Under these exercise conditions, the body produced stronger co-contractions to enhance dynamic stability, thereby reducing bilateral differences (Gabriel et al., 2006).

Similarly, the joint moments' results showed an interaction effect of lower extremity across sides and loading conditions, which indicated that the influence of different sides of the lower extremity joint moments of the barbell squat was moderated by loading conditions in SL and DOL. The dominant side joint moments were higher than those of the non-dominant side, whereas in the NDOL, the difference between the two sides of the lower extremity decreased, with a smaller difference of 10% compared to 5%. This suggests that, when an offset load occurs on one side of the barbell, the lower extremity joint moments on that side increase. Asymmetric loading is an important factor affecting biomechanical changes in the lower limb (Jeong et al., 2016). In barbell squats, there is an increased load on one side of the limb, resulting in a disruption of the body's equilibrium, and there is an increased activation and co-contraction of the muscles on one side of the limb, which increases joint moments to maintain the stability of the overall movement (Flaxman et al., 2017). The results of the present research are similar to those of previous studies, in that there was bilateral asymmetry in the participants' joint moments in the lower extremities during symmetrically loaded barbell squats (Flanagan and Salem, 2007; Kobayashi et al., 2010). In addition, the results showed that the *SI_M_* was significantly negatively correlated with the offset load in the NDOL. The *SI_M_* decreased as the offset load increased. Specifically, the hip and the knee were reduced by 5.6% and 1.9%, respectively, in 5% NDOL, whereas they decreased by 7.5% and 5%, respectively, in 10% NDOL. When the offset load increased, one side of the limb produced greater joint moments. This pattern suggests that when the load increases, the limb requires a greater joint moment to maintain the body posture during movement (Sohn and Koo, 2023). This change may be beneficial in populations with bilateral strength asymmetries, in which NDOL results in similar joint moments bilaterally in the lower extremities, potentially reducing bilateral strength differences.

Nonetheless, this study has some limitations. In the present study, participants were acclimatized to the barbell squat under an asymmetric load one week before the start of the experiment to eliminate the effects of learning; however, only the acute effects of the squat under asymmetric load were tested. Therefore, the long-term effects of barbell squats on asymmetrical loads remain unclear. The sample size of this study was small; a larger sample size would have provided more accurate results. Further research should examine the application of asymmetric load barbell squats in different populations, particularly in athletes with varying exercise demands and rehabilitation populations. Kinematics were not explored, instead this study tightly controlled the speed and posture of the movements. In addition, we did not assess the prime mover muscles such as the vastus lateralis and adductor magnus, nor did we assess the trunk or core muscles, which should be explored in future studies.

### Practical Implications

The results of this study provide a practical reference for athletes to conduct physical training and for coaches to design training programs. Asymmetric load barbell squat training can be an important modality for enhancing strength while reducing bilateral strength asymmetry in the lower extremities. In sports such as skiing, swimming, and track and field, where the use of one side of the body is excessive or more dependent on one side of the body for the execution of movement techniques, and where bilateral strength balance is emphasized, asymmetric load training may be a useful addition to fitness training to reduce the risk of injury and improve performance. In specific applications, athletes should always be reminded to maintain the stability and balance of the spine and pelvis. The total and offset loads should be regulated according to the strength level of athletes, and the use of protective measures is recommended to ensure the safety of athletes' training when large offset loads are applied.

## Conclusions

The 5% NDOL and 10% NDOL barbell squats reduced the differences in joint moments and muscle activity between the dominant and non-dominant sides of the lower extremity, with 10% NDOL being more favorable and bilateral differences diminishing with increasing amounts of the offset load. The non-dominant side offset load barbell squat can be used as an important training modality to balance the strength between the lower extremities bilaterally, prevent sports injuries, and improve performance.
